# Prognostic utility of the breast cancer index and comparison to Adjuvant! Online in a clinical case series of early breast cancer

**DOI:** 10.1186/bcr3038

**Published:** 2011-10-14

**Authors:** Rachel C Jankowitz, Kristine Cooper, Mark G Erlander, Xiao-Jun Ma, Nicole C Kesty, Hongying Li, Mamatha Chivukula, Adam Brufsky

**Affiliations:** 1 Department of Medicine, Division of Hematology/Oncology, UPMC, University of Pittsburgh Cancer Institute, 300 Halket Street, Suite 4628, Pittsburgh, PA 15213, USA; 2 Department of Biostatistics, UPMC, University of Pittsburgh Cancer Institute, 300 Halket Street, Suite 4628, Pittsburgh, PA 15213, USA; 3 bioTheranostics, Inc., 9640 Towne Center Drive, Suite 200, San Diego, CA 92121, USA; 4 Department of Pathology, UPMC, 300 Halket Street, Suite 4628, Pittsburgh, PA 15213, USA

## Abstract

**Introduction:**

Breast Cancer Index (BCI) combines two independent biomarkers, HOXB13:IL17BR (H:I) and the 5-gene molecular grade index (MGI), that assess estrogen-mediated signalling and tumor grade, respectively. BCI stratifies early-stage estrogen-receptor positive (ER+), lymph-node negative (LN-) breast cancer patients into three risk groups and provides a continuous assessment of individual risk of distant recurrence. Objectives of the current study were to validate BCI in a clinical case series and to compare the prognostic utility of BCI and Adjuvant!Online (AO).

**Methods:**

Tumor samples from 265 ER+LN- tamoxifen-treated patients were identified from a single academic institution's cancer research registry. The BCI assay was performed and scores were assigned based on a pre-determined risk model. Risk was assessed by BCI and AO and correlated to clinical outcomes in the patient cohort.

**Results:**

BCI was a significant predictor of outcome in a cohort of 265 ER+LN- patients (median age: 56-y; median follow-up: 10.3-y), treated with adjuvant tamoxifen alone or tamoxifen with chemotherapy (32%). BCI categorized 55%, 21%, and 24% of patients as low, intermediate and high-risk, respectively. The 10-year rates of distant recurrence were 6.6%, 12.1% and 31.9% and of breast cancer-specific mortality were 3.8%, 3.6% and 22.1% in low, intermediate, and high-risk groups, respectively. In a multivariate analysis including clinicopathological factors, BCI was a significant predictor of distant recurrence (HR for 5-unit increase = 5.32 [CI 2.18-13.01; *P *= 0.0002]) and breast cancer-specific mortality (HR for a 5-unit increase = 9.60 [CI 3.20-28.80; *P *< 0.0001]). AO was significantly associated with risk of recurrence. In a separate multivariate analysis, both BCI and AO were significantly predictive of outcome. In a time-dependent (10-y) ROC curve accuracy analysis of recurrence risk, the addition of BCI+AO increased predictive accuracy in all patients from 66% (AO only) to 76% (AO+BCI) and in tamoxifen-only treated patients from 65% to 81%.

**Conclusions:**

This study validates the prognostic performance of BCI in ER+LN- patients. In this characteristically low-risk cohort, BCI classified high versus low-risk groups with ~5-fold difference in 10-year risk of distant recurrence and breast cancer-specific death. BCI and AO are independent predictors with BCI having additive utility beyond standard of care parameters that are encompassed in AO.

## Introduction

Approximately 75% of women with early-stage, estrogen receptor-positive (ER^+^), lymph node-negative (LN^-^) breast cancer treated with endocrine therapy do not develop distant relapse of disease [1]. Adjuvant endocrine therapy alone may be sufficient for the majority of ER^+ ^LN^- ^patients. However, because it is difficult to identify those ER^+ ^LN^- ^patients with a low risk for recurrence, many patients are subjected to unnecessary chemotherapy and the potential for increased toxicity without added benefit [1]. Currently, within the early-stage hormone receptor-positive patient population, treating physicians rely heavily on traditional clinicopathological criteria such as age, tumor size, and tumor grade to better quantify individual risk and direct systemic therapy.

Adjuvant! Online (AO) is a web-based actuarial tool that incorporates such criteria in order to predict patient outcome at 10 years on the basis of data from the Surveillance, Epidemiology, and End Results (SEER) registry and therapeutic benefit on the basis of the Oxford overviews of randomized clinical trials [2]. The AO model was developed in the US and validated with a Canadian cohort [2] but is subject to a number of limitations. For example, individualized AO estimates of recurrence risk are sensitive to variability in comorbidity assessment [3], and its (Adjuvant! Online) estimates of recurrence are not truly individualized, because they are based on data incorporated into 'binned' categories (tumor size, nodal status, and so on) [4].

Genomic-based assays are standardized, reproducible prognostic tools that have the potential to assess recurrence risk beyond standard clinicopathological parameters [5]. The Breast Cancer Index (BCI), a gene expression-based signature, is a continuous risk prediction model that combines the gene expression profiles of the HOXB13/IL17BR ratio (H:I) and the Molecular Grade Index (MGI) [6,7]. BCI was developed and tested within the previously conducted prospective Stockholm trial of low-risk, ER^+ ^LN^- ^women randomly assigned to tamoxifen versus no therapy [6,8]. Within this cohort, BCI is a highly significant prognostic tool, identifying more than 50% of patients as low-risk [6]. Although H:I and MGI both predict clinical outcome in patients with ER^+ ^LN^- ^breast cancer, these biomarkers measure different components of the underlying tumor biology. H:I is linked to dysfunctional estrogen signalling in breast cancer [9], and MGI is a five-gene signature that recapitulates tumor grade [7]. In a previous study, the combination of H:I and MGI was demonstrated to be superior to either index alone in predicting breast cancer recurrence risk in ER^+ ^LN^- ^breast cancer [7]. The aims of the present study were to validate BCI in a clinical case series and to compare the prognostic utility of BCI with that of AO.

## Materials and methods

### Study population and tumor samples

Patients were identified by a search of the Cancer Registry Data at the UPMC University of Pittsburgh Cancer Institute, a large database that includes comprehensive demographic and clinical outcome data on patients with breast cancer. Eligible patients included all subjects who had ER^+ ^(10% or more 'nuclear' staining of the tumor cells), LN^- ^invasive breast cancer diagnosed between 1990 and 1999 and who had received adjuvant tamoxifen. In addition, patients were included in the study only if there was an associated formalin-fixed, paraffin-embedded (FFPE) tissue block (or blocks) from the time of original diagnosis in order to confirm ER status and tumor grade. This study was approved by the Institutional Review Board at the University of Pittsburgh. In accordance with the approval, informed consent from the patients was not required.

A hematoxylin and eosin (H&E) slide for each case was reviewed to confirm tumor grade by using the Nottingham grading system to include tubules, nuclear grade, and mitosis. To confirm ER status, one representative tumor block was selected for immunohistochemistry (IHC) analysis. IHC was performed on the selected FFPE tissue blocks with pre-dilute rabbit monoclonal antibodies directed against ER alpha (SP1; Ventana, Tucson, AZ, USA), and the recommended protocols of the manufacturers were followed as specified. A tumor was considered 'ER-positive' if there were 10% or more 'nuclear' staining of the tumor cells.

### Breast Cancer Index and Adjuvant! Online calculation

A representative block for each case was processed at UPMC, and unstained sections were sent to bioTheranostics, Inc. (San Diego, CA, USA) for BCI analysis. All laboratory and raw data analyses were subsequently completed at bioTheranostics, Inc. without knowledge of clinical outcome. For each case, an H&E slide was generated at bioTheranostics, Inc. and examined to confirm 40% content of invasive cancer. Macrodissection of invasive tumor cells was completed. RNA was extracted and a real-time reverse transcription-polymerase chain reaction assay for BCI was performed as previously described [7,10]. The genes analyzed were HOXB13, IL17BR (HOXB13:IL17BR (H:I) index), BUB1B, CENPA, NEK2, RACGAP1, RRM2 (Molecular Grade Index), ACTB, HMBS, SDHA, and UBC (reference genes). Primer and probe sequences for these genes were the same as previously described [7,11]. Raw polymerase chain reaction data were collected and subsequently the BCI algorithm was calculated and categorical risk was assigned for each case: low risk, BCI of less than 5; intermediate risk, BCI of from at least 5 to less than 6.4; high risk, BCI of at least 6.4 [6]. Predefined BCI scores were sent to UPMC, and, once they were submitted, associated clinical outcome for each patient was linked with the respective BCI risk score. AO scores for risk of recurrence and mortality were calculated on the basis of the online tool Adjuvant! for Breast Cancer version 8.0 [12]. Clinicopathological data, which included age, tumor size, tumor grade, ER status, and nodal status, were collected for each patient by a third-party, honest broker, who then provided the de-identified outcome information to the clinical investigators. The clinicopathological data and treatment for individual patients were then inputted online, and AO 10-year predictions for recurrence and mortality were obtained.

### Statistical analysis

#### Breast Cancer Index validation

The primary endpoint was distant recurrence-free survival (defined as the time from diagnosis to the time of first distant metastasis). Secondary endpoints included breast cancer-specific survival (defined as the time from diagnosis to the time of a breast cancer-related death), overall survival (defined as the time from diagnosis to the time of death from any cause), and 10-year rates of distant metastasis, all-cause mortality, and breast cancer-specific mortality. For time to distant recurrence, event times were censored at the time of a second primary or at the time of a local recurrence or at the last follow-up time for those with no event. For time to breast cancer death, event times were censored at the time of a second primary or at the time of non-breast cancer death or at the last follow-up time for those alive. For time to death by any cause, the event times were censored at the last follow-up time for those alive.

Chi-square tests for independence were performed to assess the association between categorical variables. Distant recurrence-free survival, overall survival, and breast cancer-specific survival and the 10-year rates of distant recurrence, all-cause mortality, and breast cancer-specific mortality were estimated for the population from Kaplan-Meier survival curves. Cox models were used to assess whether BCI as a continuous risk model provided prognostic information independently of traditional clinical and histological parameters (age, tumor size, tumor grade, and so on). The hazard ratio (HR) for the continuous BCI score was calculated relative to an increment of 5 BCI units.

#### Breast Cancer Index comparison with Adjuvant! Online

To compare BCI with AO, the continuous risk model of BCI was used as previously described [6]. AO predicts recurrence defined as the reappearance of breast cancer at any site (local, contralateral, or distant) and breast cancer mortality (10-year follow-up), both of which are estimated and derived from total survival analyses of SEER data [13]. To compare the prognostic abilities of AO and BCI, the 10-year predicted risk of any recurrence or mortality was calculated from AO and evaluated in conjunction with the predicted risk of distant recurrence by BCI. Given the differences in defining breast cancer-specific death between BCI and AO, breast cancer survival with AO was compared with breast cancer-specific survival and overall survival with BCI. BCI and AO are scored on different numerical scales: 0 to 10 for BCI and 0 to 100 for AO. To more accurately compare BCI with AO, HRs were calculated relative to an increment of their interquartile ranges (2.936 for BCI and 5 for AO). All statistical procedures - with the exception of the receiver operating characteristic (ROC) analysis, which was performed in R (version 2.13.0) - were conducted with the statistical software SAS (version 9.2; SAS Institute Inc., Cary, NC, USA).

The predictive accuracy of the BCI score was determined by using a time-dependent ROC curve method for censored survival data [14,15]. A Cox model with selected covariates was fit, and the predicted values were used to generate time-dependent sensitivity and specificity and the corresponding ROC curve at each observed event time. The area under the curve was plotted over time to assess the predictive accuracy of the model in distinguishing subjects who have an event before time *t *from those who do not. Models containing only the AO score versus models containing the AO plus the BCI scores were generated to quantify the impact that BCI had on the predictive accuracy for the outcomes of time to distant recurrence and time to breast cancer-specific death for all subjects and for subjects treated with tamoxifen alone.

To determine the accuracy of risk assessment by BCI and AO over a period of 10 years, we completed a global concordance summary (integrated area under the curve, or iAUC), which is a measure of agreement between survival time and predicted risk over a period of 10 years of follow-up [14,15]. The concordance measure estimates the probability that, for two randomly chosen individuals, the subject with the shorter survival time also has the larger risk score. A model with perfect agreement would have a value close to 1, whereas a value of 0.5 is no better than random chance.

## Results

### Patient characteristics and Breast Cancer Index distribution

Of the 386 patients who were identified in the UPMC database for analysis and who met the eligibility criteria (ER^+ ^LN^- ^tamoxifen-treated), 265 had paraffin tissue blocks available for assessing BCI. Patient characteristics are shown in Table 1. Overall, the median time of follow-up for all patients was 10.3 years, the rate of distant recurrence was 15%, and mortality rates were 11% and 15% for breast cancer-specific and all-cause mortality, respectively. As this was a clinical case series, treatment was not uniform. Of the 265 patients, 85 (32%) received adjuvant chemotherapy in addition to adjuvant tamoxifen, whereas 180 (68%) were treated with tamoxifen alone. Non-recurrence and recurrence rates between these two treatment groups were not significantly different (*P *~0.2), and the chemotherapy-treated group consisted of patients who were younger (51% were younger than 50 years old; *P *< 0.0001) and who had higher tumor grade (*P *= 0.04) and larger tumors (*P *< 0.0001) in comparison with the group treated with tamoxifen alone.

**Table 1:** Patient characteristics

Description	Patients without chemotherapy	Patients with chemotherapy	*P *value^a^	All patients
Number	180	85		265
Age, years				
Median (range)	60 (34, 81)	49 (25, 70)		56 (25, 81)
≥ 50	143 (79%)	42 (49%)	< 0.0001	
Tumor grade			0.040	
1	53 (29%)	13 (15%)		66 (25%)
2	101 (56%)	55 (65%)		156 (59%)
3	26 (14%)	17 (20%)		43 (16%)
Tumor size				
Mean (standard deviation)	1.4 (0.9)	2.2 (1.4)		1.7 (1.2)
≥ 2 cm	32 (18%)	45 (53%)	< 0.0001	77 (29%)
Radiation treatment			0.5073	
Yes	150 (83%)	68 (80%)		218 (82%)
No	30 (17%)	17 (20%)		47 (18%)
Recurrence event			0.199	
No recurrence	137 (76%)	66 (78%)		203 (77%)
Locoregional/Contralateral recurrence	12 (7%)	1 (1%)		13 (5%)
Distant recurrence	25 (14%)	16 (19%)		41 (15%)
Second primary	6 (3%)	2 (2%)		8 (3%)
Median (range) follow-up, years	10.2 (2.3, 18.0)	10.3 (2.9, 17.3)		10.3 (2.3, 18.0)
Breast cancer-specific death events	21 (12%)	9 (11%)		30 (11%)
All-cause death events	29 (16%)	11 (13%)		40 (15%)

^a^Comparing tamoxifen alone versus tamoxifen plus chemotherapy groups.

For all 265 patients, the pre-defined categorical risk stratification by BCI resulted in more than half of the patients (55%) stratified as low risk with 21% and 24% as intermediate and high risk, respectively (Table 2). For the 180 patients treated with tamoxifen alone, BCI stratified 59%, 19% and 22% of patients as low, intermediate and high risk respectively (Table 2).

**Table 2:** Kaplan-Meier estimates of the rate of event for Breast Cancer Index risk groups

		Rate at 10 years (standard error)		
Risk category	Percentage of patients (*n *= 265)	Distant metastasis	All-cause mortality	Breast cancer-specific mortality
Low	54.7%	6.6% (2.2%)	6.7% (2.2%)	3.8% (1.7%)
Intermediate	21.1%	12.1% (4.8%)	8.0% (3.9%)	3.6% (2.5%)
High	24.2%	31.9% (6.1%)	28.2% (5.9%)	22.1% (5.4%)

### Association of Breast Cancer Index with patient outcome

The 10-year rates of distant metastasis, all-cause mortality, and breast cancer-specific mortality are shown in Table 2. For all patients, the 10-year rates of distant metastasis-free survival were 93.4%, 87.9%, and 68.1% for low-, intermediate-, and high-risk BCI categories, respectively (Table 2). The overall survival rates were 93.3%, 92.0%, and 71.8% and the breast cancer-specific survival rates were 96.2%, 96.4%, and 77.9% for the low-, intermediate-, and high-risk BCI groups, respectively.

For patients treated with tamoxifen alone, the 10-year rates for recurrence and mortality were similar. The 10-year rates of distant metastasis-free survival were 94.7%, 90.9%, and 66.8% for low-, intermediate-, and high-risk groups, respectively. The rates for overall survival were 92.8%, 90.0%, and 72.2% and the rates for breast cancer-specific survival were 96.7%, 97.1%, and 77.4% for the low-, intermediate-, and high-risk groups, respectively.

Proportional hazards models for outcome were performed with and without BCI and included the known prognostic clinical variables of age, tumor size, tumor grade, and treatment (Tables 3, 4, 5). BCI was evaluated as a continuous variable as previously developed in a retrospective analysis of the Stockholm trial [6]; the HR for recurrence and mortality was calculated relative to an increment of 5 BCI units, which is half the range of BCI.

**Table 3:** Cox proportional hazards model for likelihood of distant recurrence

	Hazard ratio (95% CI)	*P *value
Multivariate without BCI		
Age, ≥ 50 versus < 50	1.46 (0.70-3.05)	0.317
Tumor size, ≥ 2 cm versus < 2 cm	1.84 (0.94-3.58)	0.074
Tumor grade, reference grade 1		0.006
Grade 2	3.60 (1.08-12.03)	0.038
Grade 3	7.04 (2.00-24.80)	0.002
Treatment^a^	1.13 (0.56-2.28)	0.742
Multivariate with BCI		
Age, ≥ 50 versus < 50	1.34 (0.64-2.82)	0.444
Tumor size, ≥ 2 cm versus < 2 cm	1.69 (0.87-3.28)	0.124
Tumor grade, reference grade 1		0.009
Grade 2	3.73 (1.11-12.54)	0.033
Grade 3	6.77 (1.92-23.81)	0.003
Treatment^a^	0.99 (0.49-2.03)	0.985
BCI^b^	5.32 (2.18-13.01)	0.0002
Multivariate with only BCI and AO		
Adjuvant! Online^c^	1.42 (1.16-1.74)	0.0007
BCI^c^	2.47 (1.50-4.07)	0.0004

^a^Tamoxifen alone versus tamoxifen plus chemotherapy groups. ^b^Hazard ratio calculated for Breast Cancer Index (BCI) relative to an increment of 5 units. ^c^Hazard ratio calculated for BCI and Adjuvant! Online (AO) relative to increments of their interquartile range (2.936 for BCI and 5 for AO). CI, confidence interval.

**Table 4:** Cox proportional hazards model for all-cause mortality

	Hazard ratio (95% CI)	*P *value
Multivariate without BCI		
Age, ≥ 50 versus < 50	2.77 (1.16-6.58)	0.021
Tumor size, ≥ 2 cm versus < 2 cm	1.24 (0.62-2.48)	0.541
Tumor grade, reference grade 1		0.004
Grade 2	1.96 (0.73-5.21)	0.180
Grade 3	4.81 (1.71-13.57)	0.003
Treatment^a^	0.99 (0.46-2.14)	0.981
Multivariate with BCI		
Age, ≥ 50 versus < 50	2.62 (1.10-6.28)	0.031
Tumor size, ≥ 2 cm versus < 2 cm	1.13 (0.56-2.27)	0.728
Tumor grade, reference grade 1		0.004
Grade 2	2.02 (0.75-5.38)	0.162
Grade 3	4.86 (1.73-13.66)	0.003
Treatment^a^	0.90 (0.42-1.96)	0.796
BCI^b^	6.77 (2.80-16.41)	< 0.0001
Multivariate with only BCI and AO		
Adjuvant! Online^c^	1.45 (1.10-1.91)	0.009
BCI^c^	2.82 (1.70-4.67)	< 0.0001

^a^Tamoxifen alone versus tamoxifen plus chemotherapy groups. ^b^Hazard ratio calculated for Breast Cancer Index (BCI) relative to an increment of 5 units. ^c^Hazard ratio calculated for BCI and Adjuvant! Online (AO) relative to increments of their interquartile range (2.936 for BCI and 5 for AO). CI, confidence interval.

**Table 5:** Cox proportional hazards model for breast cancer-specific mortality

	Hazard ratio (95% CI)	*P *value
Multivariate without BCI		
Age, ≥ 50 versus < 50	2.34 (0.90-6.13)	0.082
Tumor size, ≥ 2 cm versus < 2 cm	1.36 (0.61-3.02)	0.451
Tumor grade, reference grade 1		0.008
Grade 2	8.08 (1.07-60.88)	0.043
Grade 3	18.22 (2.33-142.53)	0.006
Treatment^a^	1.09 (0.45-2.61)	0.850
Multivariate with BCI		
Age, ≥ 50 versus < 50	2.23 (0.85-5.87)	0.103
Tumor size, ≥ 2 cm versus < 2 cm	1.28 (0.57-2.87)	0.554
Tumor grade, reference grade 1		0.008
Grade 2	8.67 (1.14-65.73)	0.037
Grade 3	18.95 (2.42-148.48)	0.005
Treatment^a^	0.96 (0.40-2.33)	0.931
BCI^b^	9.60 (3.20-28.80)	< 0.0001
Multivariate with only BCI and AO		
Adjuvant! Online^c^	1.52 (1.11-2.08)	0.009
BCI^c^	3.27 (1.79-5.98)	0.0001

^a^Tamoxifen alone versus tamoxifen plus chemotherapy groups. ^b^Hazard ratio calculated for Breast Cancer Index (BCI) relative to an increment of 5 units. ^c^Hazard ratio calculated for BCI and Adjuvant! Online (AO) relative to increments of their interquartile range (2.936 for BCI and 5 for AO). CI, confidence interval.

When BCI was included in the model of known prognostic factors, BCI was highly significant and was associated with recurrence risk (HR = 5.32; 95% confidence interval (CI) 2.18 to 13.01; *P *= 0.0002; Table 2), all-cause mortality (HR = 6.77; 95% CI 2.80 to 16.41; *P *< 0.0001; Table 3), and breast cancer-specific mortality (HR = 9.60; 95% CI 3.20 to 28.80; *P *< 0.0001; Table 4).

### Comparison of Breast Cancer Index with Adjuvant! Online

Univariate Cox models were fit separately for both BCI and AO. AO was significantly associated with risk of recurrence (HR = 1.53; 95% CI 1.25 to 1.86; *P *< 0.0001), all-cause mortality (HR = 1.58; 95% CI 1.22 to 2.06; *P *= 0.0006), and breast cancer-specific mortality (HR = 1.68; 95% CI 1.24 to 2.23; *P *= 0.0001). BCI was also significantly associated with risk of recurrence (HR = 2.77; 95% CI 1.71 to 4.51; *P *< 0.0001), all-cause mortality (HR = 3.03; 95% CI 1.86 to 4.94; *P *< 0.0001), and breast cancer-specific mortality (HR = 3.53; 95% CI 1.98 to 6.31; *P *< 0.0001). A combined multivariate analysis with only BCI and AO showed that both independently remained significantly associated with risk of recurrence (Table 3), all-cause mortality (Table 4), and breast cancer-specific mortality (Table 5).

To determine the accuracy of risk assessment by BCI and AO over the course of a 10-year period, we completed a global concordance summary, which is a measure of agreement between survival time and predicted risk over the course of a 10-year period of follow-up (iAUC). The concordance measure estimates the probability that, for two randomly chosen individuals, the subject with the shorter survival time also has the larger risk score. A model with perfect agreement would have a value close to 1, whereas a value of 0.5 is no better than chance. For time to distant recurrence for all patients, iAUC values were 0.642 (0.586, 0.716) for models with AO only and 0.717 (0.656, 0.800) for models with AO+BCI. For the patients treated with tamoxifen alone, these probability values increased to an iAUC of 0.671 (0.589, 0.754) and 0.750 (0.659, 0.847) for models with AO only and AO+BCI, respectively.

**Figure 1:**
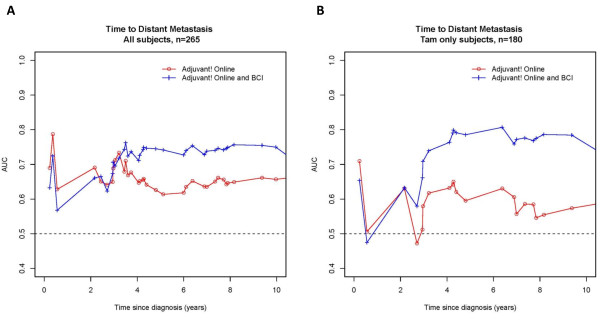
**Time-dependent receiver operating characteristic curve analysis of Adjuvant! Online (AO) and AO with Breast Cancer Index (BCI)**. Linear predictors are derived from a Cox model with AO only (red circles) and from a model with AO and BCI (blue plus) for **(a) **all subjects and for **(b) **subjects treated with tamoxifen (Tam) alone. Curves plot the area under the curve (AUC) over time and compare the accuracy of the model score to distinguish patients who will develop a distant metastasis from those who will not. The separation of the curves demonstrates the gain in predictive accuracy of including BCI in the model.

Time-dependent ROC curve analyses over the 10-year period of outcome demonstrated that, for early time points (< 4 years), the model scores provided limited differential ability (between AO versus AO+BCI) in distinguishing those patients who had the event before time *t *from those who did not. However, for time points approximately 4 years after diagnosis, the predictive accuracy for recurrence increased for models including BCI compared with models with AO only. For all patients, the minimum and maximum predictive accuracies from 4 to 10 years were 61.4% to 66.2% for AO only and 71.1% to 75.7% for AO+BCI. For patients receiving tamoxifen alone, the ranges for this time period were 54.6% to 65.0% for AO and 73.4% to 80.7% for AO+BCI (Figure 1).

## Discussion

Prognostication of individual risk for distant recurrence and death for patients with ER^+ ^LN^- ^breast cancer treated only with adjuvant endocrine therapy continues to be a challenge given that approximately 75% of these patients remain disease-free at 15 years [1]. In addition, the Oxford overview of a meta-analysis of 34,873 women reports the low recurrence rate of 2.2% per year for ER^+ ^LN^- ^patients receiving tamoxifen alone [16]. Furthermore, 12-year follow-up of 1,536 patients who had ER^+ ^LN^- ^breast cancer and who were randomly assigned to tamoxifen versus tamoxifen plus chemotherapy had recurrence-free survival rates of 79% and 89%, respectively, and overall survival rates of 83% and 87%, respectively, and absolute chemotherapy benefits of 10% for recurrence-free survival and 4% for overall survival. A large proportion of ER^+ ^LN^- ^patients are disease-free more than 10 years after diagnosis when treated with endocrine therapy alone and, therefore, could forgo chemotherapy and the associated toxicities. Prognostic indices, which identify low-risk patients, can be used to generate information to aid in the treatment decision process for determining optimal adjuvant therapy for ER^+ ^LN^- ^patients.

Early breast cancer assessment tools for prognosis such as the St Gallen breast cancer consensus guidelines and the AO assess risks and benefits associated with adjuvant therapy [13,17,18]. For AO, the selection criteria for withholding chemotherapy is based primarily on the integration of clinicopathological correlates (tumor grade, nodal status, tumor size, and ER status) and comorbidities, all of which have inherent limitations in assessment or measurement [3,4]; however, correct assessment is of major importance in order to avoid unnecessary toxic side effects associated with chemotherapy [18].

Here, we report the prognostic performance of the gene expression-based BCI within a clinical case series of patients with ER^+ ^LN^- ^breast cancer and demonstrate that BCI is a highly significant predictor of distant metastasis and death in patients treated with adjuvant tamoxifen, with or without chemotherapy. With categorical stratification, BCI identified more than 50% of the patients with low risk with a 10-year rate of recurrence of 6.6% and breast cancer-specific mortality rate of 3.8%. In a multivariate model that includes clinicopathological covariates, BCI remained a significant factor associated with recurrence risk and mortality.

Prognostic gene expression signatures have the capability to offer objective and reproducible predictive risk assessments beyond the traditional criteria used for AO to guide adjuvant treatment selection. The overall correlation between BCI and AO for individual 10-year risk of recurrence assessment was low (*r *= 0.2), and AO risk was higher for most patients. For example, of the study's 226 patients who were defined by AO as having at least a 10% risk of recurrence at 10 years, 51% were classified by BCI as low-risk, 22% as intermediate-risk, and 27% as high-risk. Actual recurrence rates for these three groups were 10%, 14%, and 34%, respectively.

Proportional hazards models that include both AO and BCI demonstrate that both factors are highly significant predictors for outcome. Furthermore, iAUC analyses demonstrate that the addition of BCI to AO increases the probability for concordance between survival time and predicted risks from 67% to 75% globally for 10-year outcome for ER^+ ^LN^- ^patients receiving tamoxifen only. Time-dependent ROC analysis enables further granulation of the additive effect of BCI to AO for predicting recurrence. The addition of BCI has its greatest accuracy benefit 4 to 10 years after diagnosis; for patients treated with tamoxifen only, BCI + AO had a recurrence prediction of 81% versus 65% for AO only.

A potential reason for the observed additive accuracy effect of BCI after 4 years may be that tumor grade, a clinicopathological covariate used in calculating AO, has its greatest prognostic strength 0 to 5 years after diagnosis; specifically, high-grade tumors are associated with increased recurrence risk. In contrast, a retrospective study (*n *= 2,838 patients) designed to identify factors associated with residual risk of recurrence (> 5 years) reported that only low-grade tumors were significantly associated with recurrence for patients receiving adjuvant therapy and disease-free for 5 years [19]. This suggests that accurate prediction of recurrence throughout the entire 10 years requires not only assessment of tumor differentiation and tumor proliferation for predicting early recurrences (that is, 0 to 5 years) but also prediction of recurrences more than 5 years by identifying the subset of low-grade or 'indolent appearing' tumors with high potential to metastasize. BCI is a score that integrates two prognostics: MGI, an index highly correlative with tumor grade [7], and H:I, which has a low correlation with tumor grade [11]. Studies are ongoing to further examine the potential differing prognostic contributions of MGI and H:I for early and late recurrences, respectively.

Current gene expression-based signatures such as the 21-gene, 70-gene, 76-gene, and 97-gene genomic grade derive a significant amount of their prognostic and predictive strength from the expression measurement of genes associated with tumor differentiation and proliferation [20-22]. For example, a multi-institutional study of 198 LN^- ^patients without systemic adjuvant therapy demonstrated that the 70-gene, 76-gene, and 97-gene genomic grade signatures had similar prognostic performance; in all three signatures, the genes controlling tumor differentiation and proliferation had the greatest prognostic discriminatory strength [20,23]. In addition, among the four different gene groups of the 21-gene signature, only the proliferative gene group is significantly associated with chemotherapy benefit in the ER^+ ^LN^- ^patient cohort randomly assigned to tamoxifen or tamoxifen plus chemotherapy [21]. Furthermore, time dependence of the prognostic performance of the 76-gene was examined over a period of 15 years to predict distant metastasis and overall survival and was compared with AO; for both risk assessments, the HRs decline over time [24]. The prognostic performance of the 21-gene signature has been compared with AO and is consistent with our findings that prediction of the risk of recurrence with the assay is independent of that with AO [25,26].

## Conclusions

Overall, this study is an independent validation of the strong prognostic performance of BCI. This study is limited by the fact that it was a retrospective, single-institution study and that results may have been biased on the basis of specimen availability and patterns of referral to the tertiary academic center. Additional studies are ongoing to validate BCI in a randomized trial population and to examine the utility of BCI to predict chemosensitivity and chemotherapy benefit. However, results from this validation study indicate that BCI identifies a large proportion of low-risk patients and is additive to AO for predicting the risk of recurrences.

## Abbreviations

AO: Adjuvant! Online; BCI: Breast Cancer Index; CI: confidence interval; ER: estrogen receptor; FFPE: formalin-fixed, paraffin-embedded; H&E: hematoxylin and eosin; H:I: HOXB13:IL17BR; HR: hazard ratio; iAUC: integrated area under the curve; IHC: immunohistochemistry; LN: lymph node; MGI: molecular grade index; ROC: receiver operating characteristic; SEER: Surveillance, Epidemiology, and End Results.

## Competing interests

ME and X-JM are employees and stockholders of bioTheranostics, Inc. and are named inventors of the HOXB13:IL17BR gene signature within an issued US patent (assignee is bioTheranostics, Inc.). NCK and HL are employees and stockholders of bioTheranostics, Inc. The other authors declare that they have no competing interests.

## Authors' contributions

AB developed the concept and contributed to the interpretation of the data. ME developed the concept, wrote the manuscript, and contributed to the interpretation of the data. X-JM developed the concept. RCJ developed the concept, collected the clinical data, wrote the manuscript, and contributed to the interpretation of the data. MC collected the clinical data. HL performed the analysis and contributed to the interpretation of the data. KC performed the analysis, wrote the manuscript, and contributed to the interpretation of the data. NCK wrote the manuscript and contributed to the interpretation of the data. All authors read and approved the final manuscript for publication.
